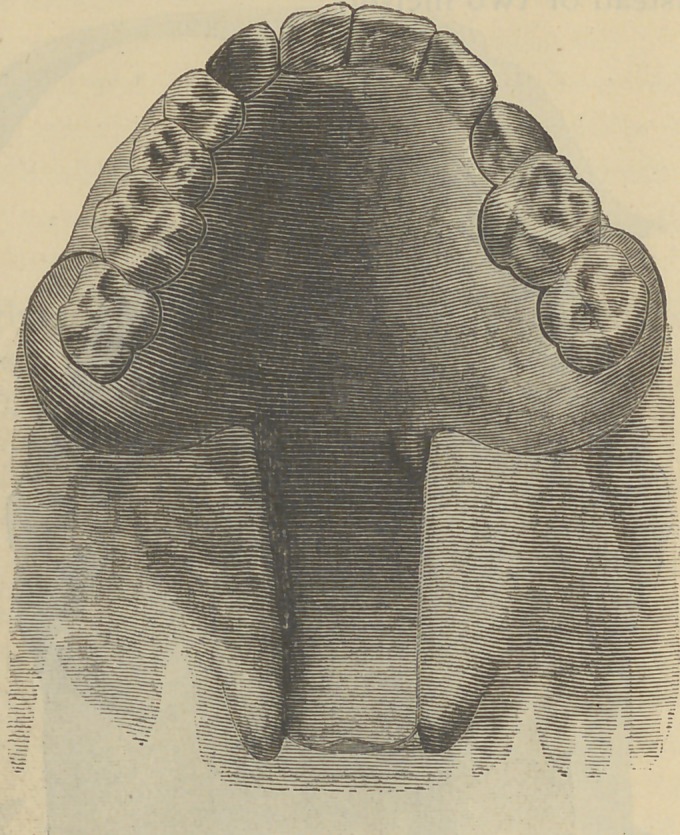# Hard Rubber Appliance for Congenital Cleft Palate

**Published:** 1878-12

**Authors:** Thomas Brian Gunning

**Affiliations:** New York


### Selections.

### HARD-RUBBER APPLIANCE FOR CONGENITAL CLEFT PLATE.

BY THOMAS BRIAN GUNNING, NEW YORK.

Alexander Petronius, in his work entitled "De Margo Gallico," and Ambrose Pare, in his book on surgery, prove that efforts to relieve those suffering from defective palate, by applying obturators, were made over three centuries ago, and the records of the last fifty years alone show that the endeavors to supplement the congenital cleft palate have resulted in the invention of mechanical appliances which in number and variety are very remarkable; yet the "Report of the International Exhibition of 1876," in referring to the one now submitted, says: "This contrivance is a very marked improvement over all previous appliances to this distressing malformation." Now, that this simple remedy was not devised earlier is owing to mistaken views as to the movement of the muscles of the pharynx and palate, both in perfect and malformed conditions, and this, notwithstanding the investigation and study of these parts by the most distinguished physiologists and surgeons.

These mistakes will be pointed out in this paper, but the literature of this malformation is already so full, especially with the recent volume on "Harelip and Cleft Palate," by Mr. Francis Mason, F. R. C. S.,* that it is not necessary to notice all varieties of congenital cleft palate, nor need attention be given to the causes of this incomplete development in fœtal structure.

* J. & A. Churchill, London, 1877.

Normal conditions will be considered first.

The constrictor muscles of the pharynx are said to be inserted into the posterior median raphe, which lies against the vertebral column, whereas they arise on that line; that is, they are fixed at this centre of the back of the pharynx, by which the inferior and middle constrictors, in deglutition, relax to allow the larynx and its support—the hyoid bone—to pass forward and open the way to the œsophagus.

The superior constrictors, which may be seen from the front of the mouth, after reaching the upper end of the raphe, are also prolonged by a fibrous aponeurosis to the basilar process of the occipital bone. They are thus firmly held up as well as back. These muscles, which form the upper part of the pharynx, pass off on each side to their insertions on the pterygo-maxillary ligament, etc. They thus inclose the tonsils, and the insertions of the muscles which arch down from the uvula.

The superior constrictor muscles, while thus firmly held at the back of the pharynx, and also at their terminations in front, where they join the attachment of the buccinators, which they resemble, are quite important, for they contract the fauces laterally and draw the tonsils and neighboring parts in, or let them out, as necessary.

The hard palate gives support along its back margin to the velum or soft palate, which is seen curving downward and ending at the uvula, which gives insertion to a pair of small muscles—the azygos uvulæ—which arise on the spine of the palate bone, and pass along the front of the velum.

The levator palati muscle comes forward and inward on each side over the concave border of the superior constrictor muscle, and spreads out in the upper surface of the velum, back of the aponeurosis of the tensor palati, which last comes down around the hamular process, and spreads out its aponeurosis to the centre of the velum and to the palate bone. The tensores palati make the velum tense; the levatores palati pull it up and back to shut off the nose, and the azygos uvulœ muscles antagonize them.

The uvula is also the centre of two distinct arches, formed by two pairs of muscles, which are separated below by the tonsils. The anterior arch is formed by the palato-glossi muscles, which are inserted into the sides of the tongue. The posterier arch is formed by the palato-pharyngei muscles, which go down, one on each side, their anterior fibres being inserted into the thyroid cartilage, while others pass around the sides and back of the pharynx.

The palato-pharyngei are not associated with the palato-glossi in constricting the isthmus of the fauces, nor does the superior constrictor act in deglutition, as supposed, its attachments making it impossible that it can press the food down the pharynx.

The form of the hard palate is such that the tongue can fit it around the inside of the teeth, as in the consonant. The back of the tongue also fits against the soft palate and avula exactly, and this closure can be maintained while the upper part of the soft palate shuts off the posterior nares. This is easily tested by pronouncing the consonant k, in which both the nose and mouth are shut off from the larynx, until the tongue leaves the palate to allow the vowel sound to come out, when only the passage to the nose is kept shut. This double closure is made even in kee, in which sound the contact for k is on the hard palate, instead of being back on the soft palate as in koo. The point of the tongue goes up in t, the back of the tongue in k, and the lower lip also goes up to form p, the upper lip and the hard palate being passive, and the soft palate nearly so, outside of its great function in respect to voice, which is to shut off the nose cavity in all sounds of speech and song except those containing m or n. At rest, the velum leaves the passage from the nose to the larynx open.

The malformed palate will now be spoken of.

Congenital cleft may be limited to the uvula, or to the front of the hard palate, or it may occupy any part of or extend through both soft and hard palate, involving the front teeth and alveolar process up into the nostrils. In nearly all cases the soft palate is seen on each side. The back of the pharynx is exposed, and appears comparatively wide and flat, although each corner holds a vertical column of tissue. which in deglutition pass rapidly toward the center of the pharynx along the surface of the constrictors, which are seen to draw strongly across; while the horizontal remnants of the soft palate, at the same time narrow the mesial gap. These vertical columns are the posterior pillars of the soft palate, which being ununited are drawn up by the levator palati of each side; but the anterior fibres of these pillars, which go to the thyroid cartilage, are seen in place aganist the tonsils. Each half of the uvula is drawn slightly up by a slip which comes from the levator, but it draws very feebly upward, the parts, except in deglutition, tending toward the sides more than up and back. Mr. Ferguson's report of a dissection, made by him, of a cleft palate in 1844, states distinctly that the superior constrictor was very full, and he also claimed for the muscle very decided forward action in deglutition, and his statement has hitherto been accepted almost without question.

The back of the pharynx is, however, in full view when the soft palate is cleft, and more especially so when the opening extends through the hard palate, but I have never seen any special action in the superior constrictor, beyond that shown in normal conditions. In 1864 I had become convinced that the superior constrictor was incapable of any action which could prevent the use of a rigid appliance to supplement the cleft soft palate, and to the present time in no case has the hard rubber palate failed to keep its place, to give entire satisfaction, and to improve the speech in a remarkable degree.

It is but justice to notice that, judging from Mr. Mason's able work referred to (p. 93), Sir William Fergusson's riper experience led to conclusions respecting the superior constrictor which are in accord with my own views, rather than with those expressed in his report of 1844.

Therefore, in brief, in view of the foregoing propositions: There being no forward action whatever of the superior constrictor muscles, a rigid plate can be worn without intermission, not only in comfort, but with improved condition of the mucous membrane, which is covered in, and of the general health, the nose being as free for breathing as in a normal condition of the parts; while the palate also enables the wearer to utilize the muscles of the cleft velume. The palate is easily made, and being of hard rubber, does not deteriorate in the mouth. It is not supported by any part of the cleft, and may thus be worn from early childhood without injury to the parts, in fact its support may even lessen the cleft.

The plate, which is held up by the teeth against the hard roof of the mouth, extends up into the cleft and thence to the back of the pharnyx near the tubercle of the atlas, the end being rounded to allow the sides of the pharynx to close in during the act of swallowing. This extension into the cleft being spread out over the soft parts on each side, the ununited muscles drawn up against it and close off the nasal cavity. The vowel sounds are therefore preserved from the resonance of the nose by the natural action of the muscles, while the nasal sounds are used when necessary, and the tongue is able to form all the lingual consonants, the stiffness of the hard rubber affording the best possible substitute for the muscular firmness of the natural soft palate. To apply this palate, a simple impression of the hard palate and teeth, as is usually taken for the setting of artificial teeth, is quite sufficient, the extension into the soft palate being made by fitting the gutta-percha pattern to the parts without subjecting the patient to the annoyance of obtaining a plaster impression of these sensitive and mobile organs. This palate is consequently so simple that any accomplished dentist can apply it, and the patient is therefore comparatively independent,

Early use of this artificial palate prevents unnatural action of the tongue, such as attempts to close the cleft with the tongue when the latter should be free to act in articulation, whether in speaking or singing.

Fig. 1 gives the upper side view of an appliance for a case in which the cleft passes through the whole length of the soft palate, but does not reach the front teeth.

Fig. 1.

Fig. 2 gives the lower front view of the plate shown in Fig. 1; when worn, the narrow part is covered on each side by the cleft soft palate, as in Fig. 4.

Fig. 2.

Fig. 3 was taken from the cast of a large cleft through both the hard and soft palate, in a patient twenty years old. The cleft in her lip had been closed in infancy; and attempts were made to close the soft palate after the cast was taken, but the parts did not unite. The case is peculiar in the absence of the bicuspid teeth and the central incisor, there being only an irregularly formed tooth on the mesial side of the canine instead of two incisors.

Fig. 3.

Fig. 4 shows the hard rubber appliance as adjusted to remedy the deformity exhibited in Fig. 3, after the wisdom teeth and the right central had been lost through decay and the malformed tooth removed.

The cut was made from an impression of the plate *in situ* after it had been worn more than four years, day and night.

Deglutition is not interfered with by cleft of the palate in adults so much as articulation or speech. It was, however, necessary to explain the movements in the pharynx and soft palate in swallowing, in order to prove that they do not interfere with a rigid but properly fitted appliance. Having shown that the constrictor muscles do not close upon the food, but that they relax to let the hyoid bone and larynx go forward, and as these views are opposed to what is laid down, it is proper to show how the food gets into the stomach.

Fig. 4.

Liquids especially are drawn into the pharynx by suction, and also pressed back by the tongue; for solid food the pressure is proportionately increased. When the food has passed into the upper part of the pharynx, it is shut in by a band or welt, consisting of the forward portion of the soft palate, continued down the sides, by the anterior pillars. The upper portion is formed by the action of the tensores palati muscles drawing their aponeuroses tight, and the palato-glossi coming into action, and continuing the curve down on each side of the tongue, at the same time assisting to draw the latter up against this arched band, or welt, by which the food is kept back.

It should be understood that the upper part of this welt is formed by the aponeuroses, at some distance in front of the uvula, so that a part of the soft palate behind the welt is left free. Through the middle of this, the azygos uvula muscles pass to the uvula, in the cente.ı of the back border or arch formed by the palato pharyngeus, curving down on each side, and known as the posterior pillars of the soft palate. These two pairs of muscles are now inactive, as the levatores palati have drawn the soft palate up behind, and closed the passage to the posterior nares, while the food is shut in at the front, as before described. At the instant this is accomplished the palato-pharyngei act, and come together behind; the levatores palati relax, and the azygos uvulæ muscles come strongly into action, and draw the uvula and the origins of the palato-pharyngei rapidly forward.

The azygos uvulœ muscles, which pass from the spine of the hard palate to the uvula, are at this time held down to the tongue by the welt or band formed by the aponeuroses before mentioned, consequently they now in acting draw the origins of the palato pharyngei forward, and down to the tongue; and as the insertions of these muscles extend down around the sides and back of the pharynx (crossing each other behind), they, in acting at this time, form a circular layer of muscular fibres, which converge from the circumference of the sides, and back of the pharynx, across to the insertion of the azygos uvulœ muscles. At this moment the muscles which arise on the inside of the chin draw the hyoid bone forcibly, the back part of the tongue is carried forward, and closes down over the epiglottis until the food falls into the œsophagus, the downward progress of the food being facilitated by the pressure of the atmosphere, which is let in by the drawing of the azygos uvulœ and the relaxation of the levatores palati muscles while the muscles of the trunk cooperate, and the food enters the stomach. It is shown that the tensores palati muscles and the paclato-glossi act in concert to form the arched band which shuts down against the tongue, and that the palato-pharyngei are not associated with the palato-glossi in constricting the isthmus of the fauces.

The foregoing explanations show that every muscle of the soft palate is active in deglutition, and that the food is effectually controlled without unreasonable action on the part of any muscle such as that generally imputed to the superior constrictor, which can not act in deglutition, as supposed, its attachments making it impossible that it can press the food down the pharynx.—*New York Med. Journal*

## Figures and Tables

**Fig. 1. f1:**
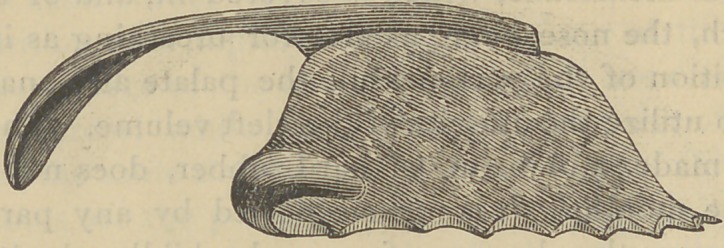


**Fig. 2. f2:**
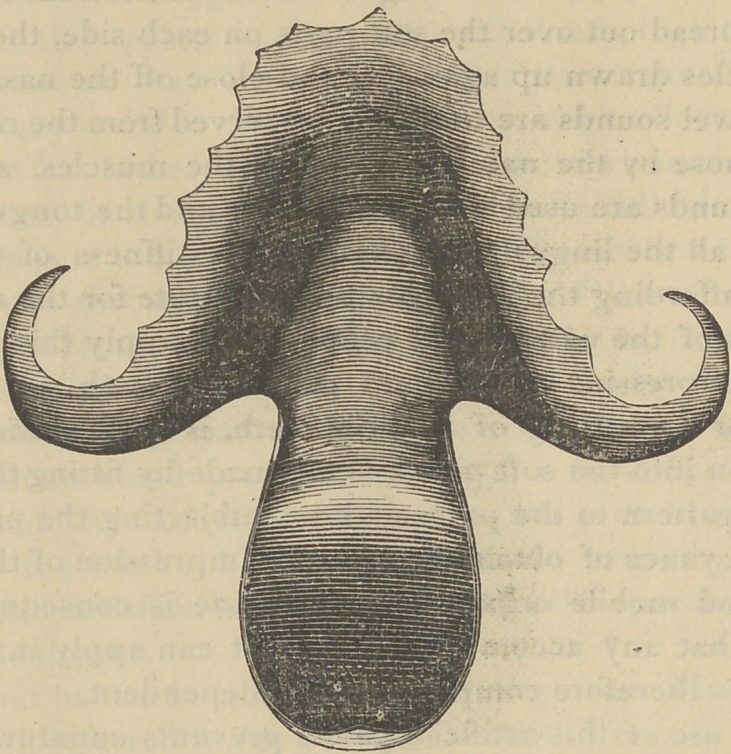


**Fig. 3. f3:**
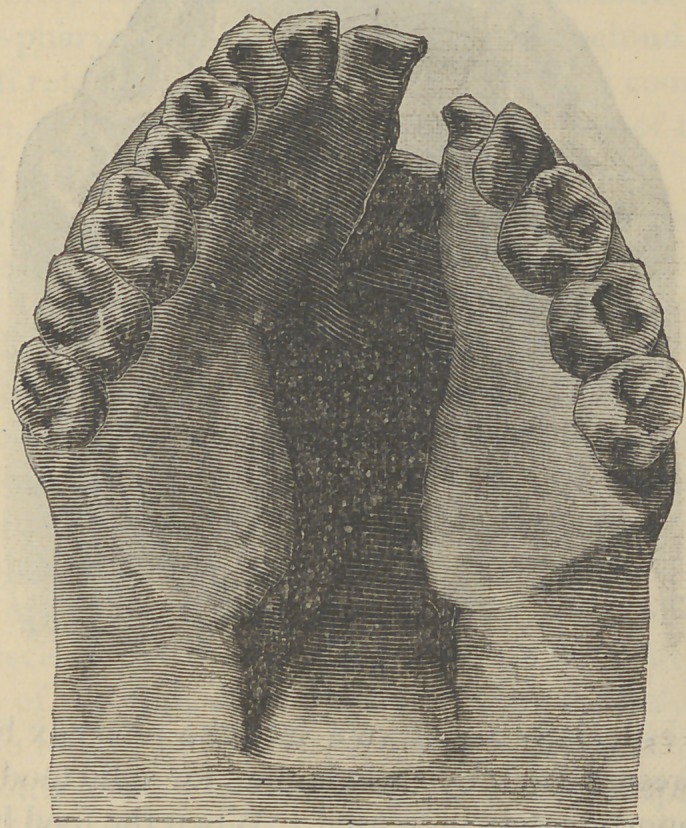


**Fig. 4. f4:**